# Using the Consolidated Framework for Implementation Research to Inform the Design of the Mobile Inspección Visual con Ácido Acético System: Mixed Methods Case Study

**DOI:** 10.2196/32577

**Published:** 2022-06-23

**Authors:** Hadley Woodruff Reid, Rae Jean Proeschold-Bell, Christina Makarushka, Katherine Dayllan Melgar Vega, Megan Huchko, Jose Jeronimo, Lavanya Vasudevan

**Affiliations:** 1 Duke University School of Medicine Durham, NC United States; 2 Duke Global Health Institute Durham, NC United States; 3 Center for Health Policy and Inequalities Research Duke University Durham, NC United States; 4 Department of Family Medicine and Community Health Duke University Durham, NC United States; 5 La Liga Contra el Cancer Lima Peru; 6 Department of Obstetrics and Gynecology Duke University Durham, NC United States

**Keywords:** cervical cancer, mobile health, Peru, colposcopy, implementation science, Consolidated Framework for Implementation Research, CFIR

## Abstract

**Background:**

There is growing evidence supporting the use of mobile health (mHealth) interventions in low- and middle-income countries to address resource limitations in the delivery of health information and services to vulnerable populations. In parallel, there is an increasing emphasis on the use of implementation science tools and frameworks for the early identification of implementation barriers and to improve the acceptability, appropriateness, and adoption of mHealth interventions in resource-limited settings. However, there are limited examples of the application of implementation science tools and frameworks to the formative phase of mHealth design for resource-limited settings despite the potential benefits of this work for enhancing subsequent implementation, scale-up, and sustainability.

**Objective:**

We presented a case study on the use of an implementation science framework in mHealth design. In particular, we illustrated the usability of the Consolidated Framework for Implementation Research (CFIR) for organizing and interpreting formative research findings during the design of the mobile Inspección Visual con Ácido Acético (mIVAA) system in Lima, Peru.

**Methods:**

We collected formative data from prospective users of the mIVAA intervention using multiple research methodologies, including structured observations, surveys, group and individual interviews, and discussions with local stakeholders at the partnering organization in Peru. These activities enabled the documentation of clinical workflows, perceived barriers to and facilitators of mIVAA, overarching barriers to cervical cancer screening in community-based settings, and related local policies and guidelines in health care. Using a convergent mixed methods analytic approach and the CFIR as an organizing framework, we mapped formative research findings to identify key implementation barriers and inform iterations of the mIVAA system design.

**Results:**

In the setting of our case study, most implementation barriers were identified in the CFIR domains of intervention characteristics and inner setting. All but one barrier were addressed before mIVAA deployment by modifying the system design and adding supportive resources. Solutions involved improvements to infrastructure, including cellular data plans to avoid disruption from internet failure; improved process and flow, including an updated software interface; and better user role definition for image capture to be consistent with local health care laws.

**Conclusions:**

The CFIR can serve as a comprehensive framework for organizing formative research data and identifying key implementation barriers during mHealth intervention design. In our case study of the mIVAA system in Peru, formative research contributing to the CFIR domains of intervention characteristics and inner setting elicited the most key barriers to implementation. The early identification of barriers enabled design iterations before system deployment. Future efforts to develop mHealth interventions for low- and middle-income countries may benefit from using the approach presented in this case study as well as prioritizing the CFIR domains of intervention characteristics and inner setting.

## Introduction

### Background

With the increasing number of mobile phone and data subscribers worldwide, mobile health (mHealth; ie, the use of mobile technologies for delivering health services and information) has become a global phenomenon [1,2]. In many low- and middle-income countries (LMICs), mHealth interventions have been successfully used to mitigate health system challenges, including human resource, infrastructure, and information constraints [2-4]. Despite the multitude of mHealth interventions that have been piloted in LMICs, few have ultimately been brought to scale or had lasting sustainability [5]. The reasons for this leaky pilot-to-scale pipeline are varied, but a key reason may be the failure to identify and address implementation barriers, especially during the early stages of mHealth intervention development [6,7].

Strategies to support the effective translation of evidence-based interventions to real-world settings fall within the purview of implementation science [8]. Implementation science tools and frameworks can guide the exploration of implementation factors, facilitate the contextualization of those factors, identify evaluation metrics and benchmarks, and provide clues to enable intervention scale-up and sustainability [9]. In recognition of the benefits of implementation science in the context of scaling mHealth interventions, the World Health Organization and others have published several guiding documents that incorporate implementation science principles and methodologies in mHealth intervention design, evaluation, and reporting [3,10,11]. These guiding documents emphasize best practices such as early stakeholder engagement; needs assessment; contextual adaptation of intervention components; interoperability with extant systems; and assessment of process outcomes such as intervention acceptability, fidelity, and adoption to explain intervention effectiveness or a lack thereof. However, in the current research paradigm, the assessment of implementation factors typically occurs concurrently with or after the evaluation of mHealth intervention effectiveness (eg, using hybrid effectiveness-implementation study designs) [12]. As a result, there are limited illustrations of how implementation science tools and frameworks may be used to pre-empt barriers, inform mHealth intervention design, and increase the chances of successful implementation and scale-up [13].

The formative phase of mHealth intervention design provides a novel opportunity to assess any unique technical or practical considerations that may influence implementation. These considerations may include acceptability of mHealth interventions in an LMIC setting, mobile phone literacy of end users, and the cultural appropriateness of intervention components. In addition, data may be needed on the feasibility of stand-alone mHealth interventions or the organizational paradigm shifts that are needed to support integration of mHealth into the health system, clinical workflows, and existing data collection and management mechanisms [14]. Hence, conducting formative research to thoroughly understand the implementation environment, barriers, and facilitators is critical for informing mHealth implementation, scale-up, and sustainability. Despite this need, existing implementation science tools and frameworks are rarely applied to the formative phase of mHealth design.

In this paper, we describe the formative research phase for an mHealth intervention—namely, the mobile Inspección Visual con Ácido Acético (mIVAA) system for cervical cancer screening—as a case study on the use of implementation science frameworks to identify implementation barriers and inform intervention design. We present the utility of the Consolidated Framework for Implementation Research (CFIR) as a convergent framework to organize formative research findings and highlight key implementation barriers. We further motivate the use of such an approach to elucidate and mitigate potential stumbling blocks for the success of mHealth implementation, which can be addressed before investment in pilot studies.

### The Problem and Proposed Digital Health Intervention

In Peru, cervical cancer is a significant contributor to mortality and morbidity among women of reproductive age. The incidence of cervical cancer in Peru is nearly twice the global rate (23.2 compared with 13.1 per 100,000 women) [15,16]. Evidence-based strategies such as early screening and preventative treatment can reduce morbidity and mortality from the disease [17]. Effective screening tests such as the Papanicolaou smear and, more recently, human papillomavirus DNA testing have successfully reduced the incidence of and mortality from cervical cancer in the United States and other high-income countries [18-20]. However, many of these strategies require resources (eg, laboratories) or personnel for implementation, and their widespread use in community-based prevention programs may be limited in LMIC settings. Thus, the World Health Organization recommends visual inspection with acetic acid (VIA) in situations where the capacity for human papillomavirus testing is lacking [17]. In VIA, health workers examine the cervix with the naked eye for aceto-whitening, which is a sign of precancerous lesions.

La Liga Peruana Contra el Cáncer (La Liga) is a nonprofit organization based in Lima, Peru. La Liga routinely organizes free large-scale screening campaigns using both Papanicolau smears and VIA with community outreach mobile units that travel around the Lima metropolitan area to low-income neighborhoods. However, the slow turnaround of Papanicolau smear results and lack of patient access to reliable means of transportation mean that approximately 77% of screened-positive women in community settings are lost to subsequent clinic-based follow-up. In consultation with key decision makers at La Liga, the use of teleconsultation with a mobile phone–based software platform combined with visual counseling using a patient’s cervical images was identified as a potential strategy to reduce this loss to follow-up. We hypothesized that this strategy may decrease loss to follow-up by using images of patients’ own anatomy to reinforce the need for clinic-based follow-up among women with suspected cancer or precancer and by increasing access to early preliminary diagnosis through expert feedback when available. Hence, La Liga partnered with Duke University and a Peruvian partner, Medical Innovation and Technology, for the design of the mIVAA system. The mIVAA system is a mobile phone–based telemedicine platform that permits the documentation of magnified cervical images for remote and asynchronous expert colposcopist feedback. Imaging of the cervix can be achieved with the built-in mobile phone camera or through a USB-connected digital colposcopy device such as a pocket colposcope [21,22]. Formative research was conducted to inform the design and implementation of the mIVAA system in La Liga’s community outreach units with the goal of identifying and mitigating implementation barriers to the maximum extent possible during the intervention development and pilot.

### Theoretical Framework

The CFIR is a framework of implementation factors often used to assess readiness for implementation and comprises 5 overarching domains: intervention characteristics, inner setting, outer setting, characteristics of individuals, and process [23]. CFIR constructs have been shown to map well to common implementation challenges in the use of digital technologies [13,24]. The CFIR has been used to evaluate the implementation of mHealth interventions in low-resource contexts, including for longitudinal assessment of 2-way SMS interventions on HIV therapy adherence and an app tracking communicable disease transmission in Uganda [25,26]. However, in both of these studies, the CFIR was used after the rollout of the mHealth intervention. The CFIR can be applied earlier in the development of interventions; however, there are limited examples of the use of this framework to guide mHealth intervention development in formative research.

We selected the CFIR for our case study as it is a well-studied and widely used framework for implementation research. Furthermore, it contains a comprehensive list of implementation factors that can be evaluated by researchers, including those with a limited background in implementation science. Finally, it also permits the description of factors related to both the general implementation context and intervention-specific factors.

Our findings demonstrate the utility of the CFIR for organizing implementation factors emerging during the formative phase of mHealth design as well as for guiding mHealth intervention development and implementation.

### Research Aims

The goal of this case study was to describe the use of the CFIR to categorize facilitators of and barriers to implementation identified during the design of the mIVAA system for cervical cancer screening in Lima, Peru. The secondary aim was to use the CFIR to inform solutions to the identified barriers before the pilot implementation of the mIVAA system.

## Methods

### Reporting

This study is reported in accordance with the consensus standards for the reporting of case studies (Table S1 in Multimedia Appendix 1) [27].

### Study Setting

This study was conducted in partnership with La Liga in Lima, Peru. The mIVAA system was designed to be used in La Liga’s community outreach units, which are staffed by a midwife (*obstetra*) and nurse technician (*técnica*). The mIVAA system comprises a digital imaging device and a telemedicine platform. The system can be used by midwives to acquire cervical images of patients in community settings and share them with expert colposcopists based in La Liga’s brick-and-mortar clinics for feedback to inform triage options. Midwives concurrently perform Papanicolau smears; however, the mIVAA system augments the naked-eye visual examination and allows for documentation via imaging.

### Study Design, Systems, and Frameworks

From April 2019 to October 2019, we conducted formative research using mixed methods to gather data on prospective implementation factors that could affect the design of the mIVAA system for cervical cancer screening. We selected the CFIR post hoc as an analytic framework for organizing the study findings for the aforementioned reasons.

### Participants

The participants consisted of health care providers and staff who were involved in the cervical cancer screening workflow at La Liga, including midwives and nurse technicians at La Liga’s 5 mobile community outreach units, colposcopists at the La Liga brick-and-mortar clinics, staff involved in patient follow-up and appointment scheduling, and La Liga administrators with decision-making authority. All eligible participants who were approached consented to take part in the study.

The same staff members who were observed in their workflow were approached for subsequent interviews, and all staff members were invited to participate in a group discussion and survey. The participants were involved in all parts of data collection as each type of data collection had a specific purpose. The observations allowed the study team to inspect the workflow in detail; the interviews solicited individuals’ thoughts on the mIVAA system and implementation context; the survey elicited general attitudes and readiness for implementation at La Liga; and, finally, the group discussions allowed for the synthesis of findings, feedback on proposed solutions, and elicitation of any residual barriers.

### Ethics Approval

This study was approved by the Institutional Review Boards of the Duke University Health System (protocol Pro00102194) and by the University of San Martín De Porres (092-2019) in Peru. All participants provided informed consent before taking part in the study and were informed of the risks and benefits of participation as well as their right to stop participating at any time.

### Data Collection

We used qualitative and quantitative methods to collect data, as described in the following sections.

#### Qualitative Data

##### Observations

Observations (n=18) of routine mobile unit and clinic workflows were conducted and recorded using a semistructured guide that emphasized documenting workflow, data collection systems, and opportunities for the integration of an mHealth telecolposcopy system with existing workflows and systems.

##### Individual Interviews and Group Discussions

Qualitative data were collected from midwives and nurse technicians (9/20, 45%) who staffed the mobile community outreach units and conducted cervical cancer screening, colposcopists (4/20, 20%) who performed diagnostic work at the La Liga brick-and-mortar clinics, staff who assisted with patient follow-up (2/20, 10%), and clinic-based administrators (5/20, 25%). Interview guides focused on the workflow of cancer screening appointments at mobile units, factors that might facilitate or inhibit the effective implementation of mHealth-supported telecolposcopy, and additional advice on incorporating telecolposcopy screening into usual clinical activities.

Written observation guides were translated from Spanish into English by bilingual study staff. Audio-recorded interviews and group discussions were first transcribed in Spanish and then translated into English by bilingual study staff. Additional bilingual study staff then read through the translated transcripts for grammar and logical flow while comparing the translations with the original Spanish.

#### Quantitative Data

We administered a cross-sectional survey to 22 participants, including midwives, nurse technicians, colposcopists, and administrative staff members. The survey included open-ended questions on the barriers to and facilitators of women receiving screening and treatment for cervical cancer, as well as the 15-item Evidence-based Practice Attitude Scale (EBPAS) examining attitudes toward the treatments, interventions, and systems related to cervical cancer [28]. The detailed methods and results of this survey have been reported previously [29].

#### Other Data

We also drew on internal organization documents and reports shared by our study partner, unpublished data from conversations with La Liga leadership, La Liga organizational materials such as descriptions of the organization mission and structure, and Peruvian telehealth legislation.

### Data Analysis

#### Qualitative Data Analysis

Qualitative data analysis occurred in 2 stages.

#### Rapid Analysis

We used rapid analysis techniques that included a systems perspective, triangulation of data, additional data collection and input from study participants (who, as noted previously, were clinic providers and staff), and an iterative process that included decision-making with study participants [30]. In the first step of the rapid analysis, members of the study team (LV, CM, and KDMV) reviewed observation notes and interview transcripts as they were collected and developed actionable outputs, including workflow diagrams, mIVAA system mock-ups, and questions for gathering feedback from study participants. Using consensus discussions with JJ and other key decision makers at La Liga, the study team arrived at potential system features that were illustrated in the mock-ups. In the second step of the rapid analysis, the study team presented the outputs to the study participants during group discussions to elicit feedback and any residual concerns or barriers related to mIVAA implementation. This feedback was used during subsequent discussions to refine the system design, and additional potential solutions were brainstormed with the study team members at La Liga and Medical Innovation and Technology to determine a course of action for system development and implementation. The rapid analysis approach was necessary to facilitate progress toward system development and pilot evaluation within the time frame of the study funding [31].

#### Content Analysis

To confirm and further elaborate on the findings from the rapid analysis approach, we conducted formal content analysis on all qualitative data, including the observation notes, individual interviews, and group discussion transcripts, in parallel to system development and pilot implementation [32]. At this stage, the CFIR framework was chosen as the organizing structure for all data. A member of the research team, HWR, conducted an initial data-driven content analysis looking for mentions of potential barriers to the implementation of mHealth interventions. She documented each potential barrier, any proposed solution, and all representative quotes for each barrier. In addition, she categorized each barrier under the relevant CFIR construct using a table in Microsoft Word. The barriers and CFIR categorization were subsequently reviewed with 2 other study team members, LV and RJP-B, and each potential barrier was discussed until consensus was reached for categorization under a CFIR construct. In addition, RJP-B evaluated the quotations, and a single representative quote was selected for each barrier. These findings were triangulated with the quantitative data as described in the following sections.

#### Quantitative Data Analysis

Summary statistics for the responses to the EBPAS were tabulated to quantify the implementation climate at La Liga and provider or staff willingness to adopt new strategies and interventions related to cervical cancer. We previously reported these findings on the EBPAS as well as other survey domains related to provider perceptions of patient-side barriers to screening and follow-up that are not included in this paper. Further details on the statistical methodology are described there [29].

#### Convergent Mixed Methods Analysis (Triangulation)

**Figure 1:**
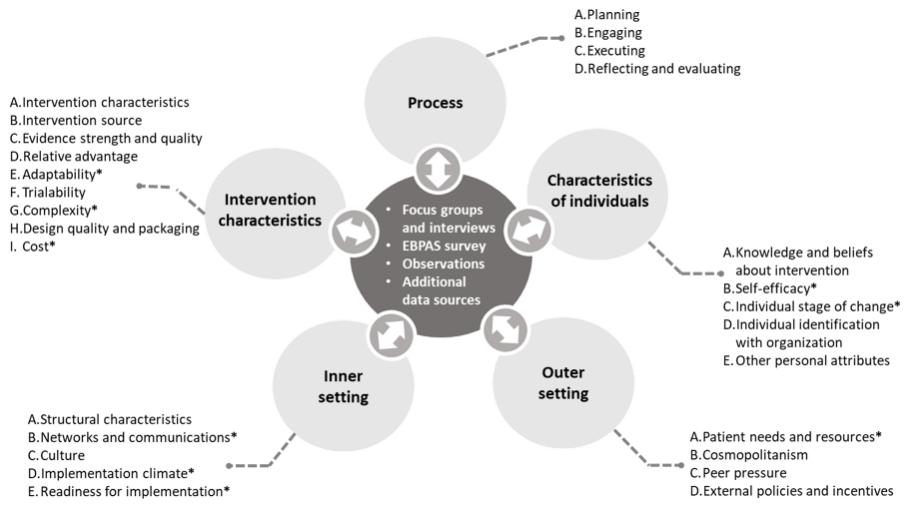
Data sources and integration of study findings using the Consolidated Framework for Implementation Research as a convergent database. *Constructs within which we identified barriers to implementation. EBPAS: Evidence-based Practice Attitude Scale.

We used both qualitative and quantitative data from our formative work to assess each construct within the CFIR domains (Figure 1). The process domain constructs will be assessed in greater detail in a subsequent pilot implementation study (see Table S2 in Multimedia Appendix 1 for a full list of domains, descriptions, and data sources). Before interpretation, we mapped qualitative and quantitative data to each CFIR construct, including facilitators of implementation as well as descriptive data on the implementation context (Table S2 in Multimedia Appendix 1). In addition, we evaluated each barrier we identified through our content analysis and mapped it to its corresponding construct in the CFIR framework (see the asterisks in Figure 1). We triangulated the findings across data sources and tallied the number of barriers identified under each construct to identify the most salient constructs for successful pilot implementation of the mIVAA system. Data interpretation is presented in a narrative format in the *Results* and *Discussion* sections of this manuscript.

## Results

### Facilitators of Implementation

Facilitators of implementation mapped broadly across the CFIR domains (Table S2 in Multimedia Appendix 1). These included many characteristics of our implementation setting—urban Peru—and our partner organization—La Liga—as well as the attitudes and perceptions of the individual La Liga staff members who would be implementing the mIVAA system. We found a receptive political environment within which La Liga was well situated to navigate local and national partnerships (outer setting). In addition, our survey data among La Liga staff showed a high willingness to accept new technologies related to cervical cancer (characteristics of individuals) [29]. However, overall, we chose to focus on barriers to implementation as these were critical to address before moving forward with the implementation of the mIVAA system.

### Barriers to Implementation

Potential barriers to implementation in our study mapped to 4 of the 5 CFIR domains; namely, intervention characteristics, outer setting, inner setting, and characteristics of individuals (Table 1). Most identified barriers fell within the domain of intervention characteristics, including the constructs of adaptability (eg, internet connectivity), complexity (eg, role definition), and cost (eg, compensation for colposcopists’ time). In addition, there were 5 potential barriers related to the domain of inner setting, with most of these related to how colposcopists would receive and process images captured with the mIVAA system and the resulting referrals. Other potential barriers included pragmatic considerations such as availability of electricity in mobile units to power digital devices and the potential impact of vibrations in the mobile units on image quality, process concerns such as time to clean and sterilize the USB-connected imaging devices between patients, scheduling an increased number of follow-up appointments in La Liga’s electronic health record system, and financial implications of the reduced number of patients able to be screened each day with the mIVAA system.

We identified 4 potential barriers to implementation that were not specific to the mIVAA system (Table S3 in Multimedia Appendix 1). These included time to sterilize speculums between clinic days (compatibility), the cost and distance of follow-up colposcopy for patients (patient needs and resources), incorrect patient phone numbers in the electronic health record system (patient needs and resources), and staff tardiness and presence at mobile units (other personal attributes). As these barriers may still affect the successful implementation of the mIVAA system, they informed data collection instruments for the pilot study.

**Table 1:** Barriers to mobile Inspección Visual con Ácido Acético (mIVAA) system implementation mapped to the Consolidated Framework for Implementation Research (CFIR) domains.

CFIR domain, construct, and potential barrier to implementation				Supporting quote or data notes				
**Intervention characteristics**								
	**Adaptability**							
		Internet connectivity			“This is a system that depends on internet connectivity, it will be as good as the internet connection we have.”			
		Vibrations and dust			“Some mobile units park on loosely packed earth and photo quality is affected by vibrations from movement in the unit.”			
		Lack of electricity in some units			“Sometimes there is no light [electricity] or water, which delays the activities, because without light the tablets or the laptops wouldn’t work.”			
	**Complexity**							
		Time for sterilization of USB-connected imaging device			“When you prepare in the squirt bottle [...] is a time that must be considered.”			
		Conflicting priorities and timing of transmitting mIVAA images			“Most of the patients came to the mobile unit at 12 noon. This makes it difficult for the midwife to send the images to the colposcopists, complete patient’s report, and other activities as soon as possible (prior to the closing time).”			
		Distinguishing results given after VIA^a^ with mIVAA from Papanicolau results			“There in that moment [with images from the digital device] you are not going to tell her, lady look there isn’t anything, come in a year. No, she [still] has to get her Pap [result] which was already taken.”			
	**Cost**							
		Financial impact of screening fewer women			“The goal of patients seen per day is 30 patients [...] With the implementation of the [mIVAA] we would have to evaluate how much the number of patients that are attended per day would decrease.”			
		Cost of colposcopists’ time			“We have to think about the budget...Right now I think it is a lie to say that a doctor will stop whatever he is doing to look at the screen and make that his priority.”			
**Outer setting**								
	**Patient needs and resources**							
		Delivery of results from mIVAA to women who have been screened	“...most of our women who are screened in the mobile units are mothers and have many duties at home, which can make it difficult for them to wait or return for their results.”					
	**External policy and incentives**							
		Legally allowable health care provision	Peruvian law states that only colposcopists may provide final review of cervical images, not midwives.					
**Inner setting**								
	**Networks and communications**							
		Communication with and availability of colposcopists	“When the colposcopist is not at La Liga, we would have to find a way in which he would be able to connect to Wi-Fi and be able to review the images and send them back to the mobile unit.”					
	**Implementation climate-relative priority**							
		Colposcopists’ desire for Papanicolau smear cytology results before evaluation	“In case the patient is scheduled for a colposcopy, we need to get Pap results as soon as possible, since some colposcopists only do colposcopy if patients have an alteration in their Pap results.”					
	**Implementation climate—compatibility**							
		Ability to correct a misdiagnosis on the telehealth platform			“What happens if I close it and maybe I made a mistake and I want to correct it?”			
		Ability to record mIVAA result in the existing WebLiga system			“Is there going to be a way to link this platform with the WebLiga system? Because when the study is done there must be a register of something.”			
	**Readiness for implementation—available resources**							
		MU^b^ space for disinfecting baths			“Mobile Unit 4 has a small space only to put the laptop [not reprocessing baths].”			
		Scheduling follow-up appointments based on mIVAA results			“There might be an accumulation of patients to follow up, which can generate more workload.”			
	**Characteristics of individuals-self-efficacy**							
		Possibility of colposcopist missing a transmitted cervical image	“The oncologist gynecologist works in different institutions. At La Liga, they only work two to three times a week with medical appointments of two hours, so I believe that it is necessary to verify the read receipt of the images.”					

^a^VIA: visual inspection with acetic acid.

^b^MU: mobile unit.

### Solutions for Implementation

The identified solutions for implementation varied (Table 2). In some cases, increased investment in infrastructure was necessary, such as providing cellular data plans to offset instances of poor internet connectivity or ensuring availability of the larger mobile units with adequate space for device setup and sterilization. Other barriers related to implementing mIVAA were able to be addressed in the subsequent pilot implementation protocol through improvements to the software interface and better role definition, such as assigning which staff member would transmit the cervical images throughout the day. A potential barrier, the ability to edit responses on the mIVAA software, could not be addressed in the pilot study because of budgetary limitations. An additional concern was that patients participating in the mIVAA intervention might mistake the results from the mIVAA screening with the final results from Papanicolau smear cytology. We did not directly address this as participants in the mIVAA intervention received the exact same counseling regarding Papanicolau smear results as women not participating in the intervention; therefore, there should not be a difference in understanding of the importance of Papanicolau smear cytology results.

**Table 2:** Solutions to identified implementation barriers by Consolidated Framework for Implementation Research (CFIR) domain.

CFIR domain, construct, and potential barrier to implementation				Solution or justification for not addressing it
**Intervention characteristics**				
	**Adaptability**			
		Internet connectivity	Providing phones with cellular plans to minimize reliance on internet connection in the mobile community outreach units. In addition, the mIVAA^a^ app can be used offline to collect data; however, image transmission to colposcopists requires network connectivity.	
		Vibrations and dust	Providing tripods to enhance camera stability. Discouraging entry or exit of mobile unit while a photo is being taken to minimize vibrations.	
		Lack of electricity in some units	Providing an external phone battery pack. Phones can be used as a light source and provide power for the imaging device via USB.	
	**Complexity**			
		Time for sterilization of USB-connected imaging device	Providing more than one imaging device (pocket colposcopes) per mobile unit to alternate between sterilization and use. Ability to use cell phone camera for image acquisition in the event that the pocket colposcopes are not ready for use.	
		Conflicting priorities and timing of transmitting mIVAA images	The midwife is asked to transmit images during the wait time between patients. To streamline data entry, the user interface of mIVAA is designed to be similar to the WebLiga system, and redundancy in data entry is minimized by using pictures of paper records.	
		Distinguishing results given after VIA^b^ with mIVAA from Papanicolau results	Midwives continue to provide usual information to women on how to collect Papanicolau results.	
	**Cost**			
		Financial impact of screening fewer women	Communicating financial impact to La Liga leaders and obtaining buy-in for lowering target recruitment to 20 patients per day during the pilot study.	
		Cost of colposcopists’ time	Identifying and recruiting colposcopists willing to participate in the study with compensation provided for time spent reviewing study images.	
**Outer setting**				
	**Patient needs and resources**			
		Delivery of results from mIVAA to women who have been screened	Adding a WhatsApp notification to colposcopists when new records are available for review to allow for same-day turnaround of results by the midwife. Colposcopists review patient records using a mobile app on their personal phone, which typically takes 2 to 3 minutes per patient.	
	**External policy and incentives**			
		Legally allowable health care provision	Ensuring that midwife role is consistent with Peruvian guidelines and only colposcopists provide image review and diagnosis.	
**Inner setting**				
	**Networks and communications**			
		Communication with and availability of colposcopists	Adding a WhatsApp notification when new records are available for review and allowing for review of patient records using a mobile app on their personal phone.	
	**Implementation climate-relative priority**			
		Colposcopists’ desire of Papanicolau results before evaluation	Working with La Liga decision makers to allow for prioritization of study participants presenting to colposcopy in laboratory queue for assessment of Papanicolau smears.	
	**Implementation climate—compatibility**			
		Ability to correct a misdiagnosis on the telehealth platform	Not addressed in the current iteration of the mIVAA system because of budgetary limitations.	
		Ability to record mIVAA result in the existing WebLiga system	Incorporating the ability to generate a printout of the mIVAA report so it can be included in the paper medical record for each patient. La Liga is exploring options for direct data import into the WebLiga system.	
	**Readiness for implementation—available resources**			
		Mobile unit space for disinfecting baths	Identifying space in the smaller mobile community outreach unit (eg, in the closet) that could be repurposed as space for disinfecting baths.	
		Scheduling increased number of follow-up appointments based on mIVAA results	Designing workflow for scheduling follow-up appointments with La Liga’s administrative leadership and staff.	
	**Characteristics of individuals-self-efficacy**			
		Possibility of colposcopist missing a transmitted cervical image	New records pending review are added to a common list allowing any colposcopist to claim and review the record. If the colposcopist does not review within 10 minutes of opening a record, the record is returned to the common list allowing other colposcopists to staff the case.	

^a^mIVAA: mobile Inspección Visual con Ácido Acético.

^b^VIA: visual inspection with acetic acid.

## Discussion

### Principal Findings

Our study describes the use of the CFIR framework to identify and organize implementation barriers in the mHealth design phase. In our case study, formative research activities informed changes to system design based on identified barriers before piloting and evaluation. We used a mixed methods analytic approach relying on both quantitative and qualitative methods in this formative research phase. The survey data indicated that the participants were favorable to the implementation of new technology related to cervical cancer screening. The individual interview, group interview, and clinic flow observation data were triangulated as, in many cases, they pointed to the same barriers, such as the need for clear role delineation when transmitting images from the mIVAA system. However, in a few cases, a data source surfaced a unique barrier. For example, it was only through the clinic observations that we discovered the need to stabilize the device to prevent shaking from mobile clinics being parked on loosely packed earth. Using a framework such as the CFIR to organize this information is valuable for categorizing potential implementation barriers and facilitators. Previously, Westgard and Fleming [33] explored the use of the Active Implementation Frameworks to guide the design and implementation of an mHealth system for monitoring child health in the Amazon region of Peru. Although the Active Implementation Frameworks provide a comprehensive combined framework, their classification into 5 separate iterative frameworks can make them difficult to apply, especially for teams without formal implementation science expertise or limited resources for formative research. In addition, we did not use a technology-specific framework such as the technology acceptance model or the unified theory of acceptance and use of technology as we were interested in the broader implementation context and how to best integrate this intervention into the organizational structure, including external and internal influences.

The CFIR has been shown to map well onto the main challenges and findings of eHealth and mHealth research [13,25]. It was designed for use in formative research; however, assessment of the use of the CFIR has shown that this tool has been mainly applied to studies already in the pilot phase of implementation or beyond [24,26,34-36]. In the rare instances where it has been used before the pilot of an intervention, it has been useful in identifying and addressing barriers to implementation in an efficient manner [37]. We selected the CFIR post hoc as a convergent database for the qualitative and quantitative data collected during our formative research and found it to be a comprehensive framework for organizing formative research data. Using the structured constructs of the CFIR allowed us to identify areas that will require additional data collection to ensure future sustainability in the eventual scale-up of the mIVAA system.

Formative research is an iterative process that both informs the constructs of the CFIR domains and serves to strengthen them by formalizing stakeholder input and providing a discussion point for brainstorming solutions. We were able to refer to Tables 1 and 2, created through the use of the CFIR domains and constructs, during the rollout of the subsequent pilot study and ensure that the identified barriers were appropriately addressed. The use of a framework to guide formative research could be a time- and cost-saving measure and provide a systematic mechanism to ensure that facilitators are taken advantage of and barriers are addressed before rollout. However, it is important to recognize that there may be financial implications to addressing barriers before implementation (eg, purchase of additional equipment and updating of software) that should be accounted for by the implementing institution when budgeting for formative research and pilot studies.

In our study, the CFIR framework revealed a conducive implementation climate and readiness for implementation. There was a strong shared understanding of the need for an intervention to reduce loss to follow-up for women after cervical cancer screening as well as significant investment on the part of La Liga in terms of staff, space, and clinic time. In addition, the constructs under the domain of characteristics of individuals were favorable for implementation based on the survey data collected on provider and staff attitudes toward innovation in cervical cancer screening.

We found that the constructs under the domains of intervention characteristics and inner setting were most likely to elicit potential barriers that could be addressed before pilot implementation, which is similar to the findings of the limited previous work using the CFIR in a preimplementation setting [38]. The formal process of formative data collection was the least applicable to the domain of outer setting, which mainly describes the political and organizational environment surrounding the intervention. Political and organizational information was gathered before our group interviews and observations through conversations with La Liga leadership and desk research. A key need identified through these discussions was clear role definition for the midwives as only trained medical doctors and colposcopists are permitted to review cervical images as per Peruvian regulations. Our findings indicate that prioritizing interview and observation guides that emphasize the constructs within the domains of intervention characteristics and inner setting may maximize the study team’s ability to elicit potential barriers and address them before piloting an intervention.

### Limitations

This is a case study and may be limited by the study context, but we believe that the process is widely applicable to other work on the design and implementation of mHealth interventions. In addition, our formative work included a relatively small sample size of participants; however, given the overall size of La Liga’s organization, this included most stakeholders and representative voices from all involved staff and clinicians. All participants were recruited through La Liga, their employer, including through a study research coordinator who was also an employee of La Liga. Although the participants provided informed consent and were able to decline participation at any time, this may have exerted some influence on their decision to take part in this study. We took multiple precautions to ensure that the study data translated from Spanish were accurate in meaning and tone; however, we acknowledge that there is always a limitation in using translated transcripts as some nuance may be lost.

As a consequence of timing constraints, we were only able to use rapid analysis findings to elicit feedback from participants during the group discussions. We then validated our convergent mixed methods analysis findings with a select few stakeholders from La Liga, including KDMV and JJ. However, we emphasize that, for future work, stakeholder insight is critical, especially when conducting formative research in an LMIC setting. Owing to the post hoc selection of the CFIR as the convergent framework, we only report on implementation factors that emerged in the data. For instance, we did not collect in-depth data on constructs related to the outer setting. Future studies may benefit from structured data collection tools that a priori encompass all CFIR domains.

### Conclusions

Formative research can provide useful insights to inform eventual implementation of mHealth interventions. The CFIR framework can be used to map and prioritize potential barriers to the implementation of mHealth interventions revealed during formative work. In our experience with an mHealth-enabled cervical cancer screening device, focusing on formative work exploring constructs under the domains of intervention characteristics and inner setting elicited the most key barriers to implementation. Future mHealth studies may choose to develop data collection tools to specifically query these domains.

## Appendix

Multimedia Appendix 1 Supplemental tables describing reporting standards, facilitators of implementation, and nonintervention-specific barriers to implementation.

## Abbreviations

CFIR: Consolidated Framework for Implementation Research
EBPAS: Evidence-based Practice Attitude Scale
La Liga: La Liga Peruana Contra el Cáncer
LMIC: low- and middle-income country
mHealth: mobile health
mIVAA: mobile Inspección Visual con Ácido Acético
VIA: visual inspection with acetic acid
