# Supplementary material for: Using the Consolidated Framework for Implementation Research to Inform the Design of the Mobile Inspección Visual con Ácido Acético System: Mixed Methods Case Study
Source: JMIR Form Res. 2022 Jun 23;6(6):e32577. doi: 10.2196/32577 (PMC9264128; doi:10.2196/32577)
Supplement: Multimedia Appendix 1 [file formative_v6i6e32577_app1.docx]

**Appendix**

**Table S1: Consensus standards for the reporting of case studies**

| Reporting Item | Page # on which item was reported | Page # of justification for not reporting |
| --- | --- | --- |
| Describing the design | | |
| 1. Define the research as a case study | 4 | NA |
| 1. State the broad aims of the study | 7 | NA |
| 1. State the research question(s)/hypotheses | 7 | NA |
| 1. Identify the specific case(s) and justify selection | 4 | NA |
| Describing the data collection | | |
| 1. Describe how data were collected | 8-9 | NA |
| 1. Describe the sources of evidence used | 8-9 | NA |
| 1. Describe any ethical considerations and obtainment of relevant approvals, access and permissions | 8 | NA |
| Describing data analysis | | |
| 1. Describe analysis methods | 10-11 | NA |
| Interpreting the results | | |
| 1. Describe any inherent shortcomings in the design and analysis and how these might have influenced the findings | 16-18 | NA |
| 1. Consider the appropriateness of the methods used for the question and subject matter and why it was that qualitative methods were appropriate | 16-18 | NA |
| 1. Discuss the data analysis | 17-18 | NA |
| 1. Ensure that the assertions are sound, neither over nor under interpreting the data | 16-18 | NA |
| 1. State any caveats about the study | 19 | NA |

| **Table S2: Application of Consolidated Framework for Implementation Research Constructs to the formative research for the implementation of** **mobile Inspección Visual con Ácido Acético (mIVAA)** | | | |  |
| --- | --- | --- | --- | --- |
| **Construct** | | **Short Description** | **mIVAA System** | **Source** |
| **I. INTERVENTION CHARACTERISTICS** | | | |  |
| A | Intervention Source | Perception of stakeholders about whether the intervention is externally or internally developed. | Perception that the device/digital imaging component was developed at Duke and software developed by Peruvian start-up Medical Innovation & Technology with input from staff at partner site, La Liga Contra el Cáncer-Perú (La Liga). Throughout formative research increasing perception of shared goals. | N/A |
| B | Evidence Strength & Quality | Stakeholders’ perceptions of the quality and validity of evidence supporting the belief that the intervention will have desired outcomes. | The “components of the system” e.g. digital imaging device received FDA approval (Device ID K181034) and through initial validation studies has been shown to have comparable imaging quality to standard colposcopes in use. Midwives express that ability to show images to patients will help enforce need for follow-up. | Mueller et al. (2018), Midwife focus group |
| C | Relative Advantage | Stakeholders’ perception of the advantage of implementing the intervention versus an alternative solution. | Positive feedback from midwives and colposcopists during the design phase, including a positive perception of the efficacy of being able to show a woman pictures of her own anatomy. | Focus groups |
| D | Adaptability* | The degree to which an intervention can be adapted, tailored, refined, or reinvented to meet local needs. | Intervention adapted to fit La Liga’s clinical workflow in the mobile units and colposcopists’ availability and preferences for review changes based on stakeholder input incorporated into pilot design. | Structured observations, focus groups |
| E | Trialability | The ability to test the intervention on a small scale in the organization, and to be able to reverse course (undo implementation) if warranted. | Planned pilot study with N=100 patient participants and N=18 La Liga staff, further activities to be evaluated after pilot. | Pilot study protocol |
| F | Complexity* | Perceived difficulty of implementation, reflected by duration, scope, radicalness, disruptiveness, centrality, and intricacy and number of steps required to implement. | Detailed assessment of La Liga clinical activities conducted to minimize number and scale of additional steps required to integrate use into standard of care cervical cancer screening. | Observations, focus groups, interviews |
| G | Cost* | Costs of the intervention and costs associated with implementing the intervention including investment, supply, and opportunity costs. | No formal cost-effectiveness analysis, but costs in major categories will be documented throughout implementation. | Pilot study data (forthcoming) |
| H | Design quality and packaging | Perceived excellence in how the intervention is bundled, presented, and assembled | Positive perception of ease of use of device and software bundle in iterative design phase by midwives. Presentation and assembly will continue to be evaluated in the pilot study when users will complete the System Usability Scale as well as an individual or group interview on acceptability/use of the imaging system. | Midwife focus group, pilot study data |
| **II. OUTER SETTING** | |  |  |  |
| A | Patient Needs & Resources* | The extent to which patient needs, as well as barriers and facilitators to meet those needs, are accurately known and prioritized by the organization. | Provider perceptions of barriers and facilitators to screening and treatment for cervical cancer assessed in previous work. These included the barriers of time, including delay of receipt of Pap results and cost of follow-up appointments. The mIVAA system is viewed as a facilitator for follow-up by allowing women to be directly counseled using images of their anatomy to give a more immediate results/referral to care. | Vasudevan et al. (2020) [Provider Survey Data] |
| B | Cosmopolitanism | The degree to which an organization is networked with other external organizations. | La Liga Contra el Cáncer-Perú (La Liga) has five mobile units (MUs) traveling to communities across Lima and Callao to offer cervical cancer screening services as well as three fully operating clinics in Lima. They have strong local linkages with municipalities which support community campaigns led by the mobile units. They are affiliated with the International Union Against Cancer (UICC), the Association of Ibero-american Leagues Against Cancer (ALICC) and the American Cancer Society. La Liga works with Instituto Nacional de Enfermedades Neoplasicas  (National Institute for Neoplastic Diseases, INEN) for post-screening referrals for further treatment and collaborates with INEN on cancer related trainings on training. La Liga also maintains relationships with donor organizations locally and internationally. | La Liga organizational materials, conversations with La Liga staff, structured observations |
| C | Peer Pressure | Mimetic or competitive pressure to implement an intervention; typically because most or other key peer or competing organizations have already implemented or are in a bid for a competitive edge. | No known additional mHealth solutions using a portable colposcope for cervical cancer screening are currently being implemented in urban Peru. However, the use of mHealth for interventions across the health system has seen significant growth over the past decade in Peru. Additionally, due to COVID-19 there is increased interest at La Liga for implementing telehealth services. | Mezarina et al. (2020), unpublished conversations with La Liga leadership |
| D | External Policy & Incentives | A broad construct that includes external strategies to spread interventions, including policy and regulations (governmental or other central entity), external mandates, recommendations and guidelines, pay-for-performance, collaboratives, and public or benchmark reporting. | Intervention is aligned with national Peruvian cancer prevention and treatment initiatives such as Plan Esperanza and the Telehealth Framework Law. Midwife role is consistent with Peruvian guidelines and only colposcopist provides final review of image. Telemedicine viewed as a mechanism to reduce geographic barriers to healthcare by Peruvian Ministry of Health; ongoing investments in building telemedicine infrastructure under the National Telemedicine Framework Law. | Plan Esperanza, National Telemedicine  Framework (2016) |
| **III. INNER SETTING** | |  |  |  |
| A | Structural Characteristics | The social architecture, age, maturity, and size of an organization. | La Liga Contra El Cancer was established in 1950 as the first organization working on the prevention and detection of cancer in Peru. Comprised of central clinic staff and midwives who work in mobile screening units in close communication with central staff. | La Liga organizational materials |
| B | Networks & Communications* | The nature and quality of webs of social networks and the nature and quality of formal and informal communications within an organization. | In-person meetings were previously used for trainings and information dissemination, during the COVID-19 pandemic this has been replaced by Zoom. Leadership has regular meetings to discuss administrative issues and logistics. Email is used for formal communication with staff, and WhatsApp is used widely for quick real-time communication between mobile outposts and main clinic staff. | Midwife focus group, unpublished data from study site coordinator |
| C | Culture | Norms, values, and basic assumptions of a given organization. | La Liga has strong commitment to improve early detection of and increase knowledge about cancers in low resource populations as evidenced by their focus on mobile units for increased accessibility of care. La Liga is also committed to improving follow-up rates for women and has been pursuing administrative changes to address low follow-up rates. | La Liga organizational materials and internal reports (e.g. “Pacientes perdidas”), focus groups |
| D | Implementation Climate | The absorptive capacity for change, shared receptivity of involved individuals to an intervention, and the extent to which use of that intervention will be rewarded, supported, and expected within their organization. | In previous work administrators and providers at La Liga scored highly in the appeal of and openness to new evidence-based practices such as digital health interventions. There is a commitment and expectation from La Liga leadership to the use of the mIVAA system in mobile units. | Vasudevan et al. (2020) |
| 1 | Tension for Change | The degree to which stakeholders perceive the current situation as intolerable or needing change. | Widespread understanding among La Liga leaders and staff that current follow-up rates are unacceptable as up to 77% of women do not present for follow-up. | Internal La Liga reports |
| 2 | Compatibility* | The degree of tangible fit between meaning and values attached to the intervention by involved individuals, how those align with individuals’ own norms, values, and perceived risks and needs, and how the intervention fits with existing workflows and systems. | Measured in prior survey as willingness to adopt new interventions for cervical cancer screening and treatment. Staff perceived low divergence of new interventions from current norm. | Vasudevan et al. (2020) |
| 3 | Relative Priority* | Individuals’ shared perception of the importance of the implementation within the organization. | There is shared commitment to improve follow-up and treatment for women with pre-cancerous or cancerous lesions and a documented understanding from La Liga leadership that the mIVAA system is a way to do this. | Focus groups, internal La Liga reports |
| 4 | Organizational incentives and rewards | Extrinsic incentives such as goal-sharing awards, performance reviews, promotions, and raises in salary, as well as less tangible incentives such as increased stature or respect | Midwives express a perceived benefit to use of the mIVAA system of the ability to show patients images of their anatomy and improve their ability to counsel and form connections with women. | Focus groups |
| 5 | Goals and feedback | The degree to which goals are clearly communicated, acted upon, and fed back to staff and alignment of that feedback with goals. | Staff included in formative feedback on implementation of the mIVAA system and invested in the long-term goal of decreasing loss to follow-up. Continuing collection of feedback on use of mIVAA system during pilot implementation shared which will be shared with staff to improve use. | Focus groups, pilot study data (forthcoming) |
| 6 | Learning climate | Interrelated practices and beliefs support and enable employee and organizational skill development, learning, and growth to maximize an organization's absorptive capacity for new knowledge and methods | La Liga holds trainings at least monthly to cover both relevant clinical topics as well as administrative skills and wellness. They are currently conducted over Zoom were previously in-person. Training on use of the mIVAA system for midwives planned for implementation pilot. | Unpublished data from study site coordinator, pilot study protocol |
| E | Readiness for Implementation | Tangible and immediate indicators of organizational commitment to its decision to implement an intervention. | La Liga leadership are involved in all stages of implementation and serve as the on-the-ground champions for use of the mIVAA system. La Liga provided a letter of support for the grant application and has signed a sub-contract for supporting study implementation. | Contractual agreements and letters of support |
| 1 | Leadership Engagement | Commitment, involvement, and accountability of leaders and managers with the implementation. | Investigators from La Liga participating in the study. All levels of leadership included in the formative interviews and meetings. | Pilot study protocol, focus groups |
| 2 | Available Resources* | The level of resources dedicated for implementation and on-going operations, including money, training, education, physical space, and time. | La Liga has previously dedicated staff time and space to formative work on evaluating the mIVAA system. La Liga additionally reduced patient quotas to allow for integration of mIVAA into study flow. Partners at Duke have committed to donating the mIVAA imaging system for use and are providing financial support for the mobile unit under use and staff salaries for activities that are directly related to the pilot study. La Liga normally relies on donor funding and clinic revenue to support their screening activities. | Contractual agreements and letters of support; structured observations |
| 3 | Access to Knowledge & Information | Ease of access to digestible information and knowledge about the intervention and how to incorporate it into work tasks. | Midwives will be trained and be given a detailed manual for use of the mIVAA system and the user-facing portions of the system have been adapted to be congruent with the current electronic data collection that La Liga uses for increased ease and familiarity. The pilot will serve as an opportunity to extend hands-on familiarization of mIVAA and provide feedback/ask questions. Research staff will be on site to provide real-time technical support in the use of mIVAA during the pilot. | mIVAA user manual, pilot study data (forthcoming) |
| **IV. CHARACTERISTICS OF INDIVIDUALS** | | |  |  |
| A | Knowledge and beliefs about the intervention | Individuals' attitudes toward and value placed on the intervention, as well as familiarity with facts, truths, and principles related to the intervention. | Prior survey and qualitative feedback from providers and staff indicated openness towards and low perceived disruptiveness of new evidence-based interventions for cervical cancer screening and treatment such as the mIVAA system. | Vasudevan et al. (2020) |
| B | Self-efficacy* | Individual belief in their own capabilities to execute courses of action to achieve implementation goals. | Self-efficacy with mIVAA system expressed during focus groups. High openness to new cervical cancer interventions by La Liga staff reported in staff surveys. | Focus groups, Vasudevan et al. (2020) |
| C | Individual Stage of Change* | Characterization of the phase an individual is in, as he or she progresses toward skilled, enthusiastic, and sustained use of the intervention. | Prior survey among health workers showed high willingness to accept new interventions related to cervical cancer career and low perceived divergence of intervention from current practice. | Vasudevan et al. (2020) |
| D | Individual identification with organization | A broad construct related to how individuals perceive the organization and their relationship and degree of commitment to that organization. | La Liga reports strong employee retention. From a sample of La Liga employees working on cervical cancer related activities the average length of employment was 11.7 years with a a standard deviation of 8.2 years. | Unpublished data from study site coordinator, Vasudevan et al. (2020) |
| E | Other personal attributes | Broad construct to include other personal traits for example tolerance of ambiguity or intellectual ability. | Openness to and appeal of evidence-based interventions for cervical cancer assessed in staff and providers as above. Results showed high appeal and openness. | Vasudevan et al. (2020) |
| **V. PROCESS** | |  |  |  |
| A | Planning | The degree to which a scheme or method of behavior and tasks for implementing an intervention are developed in advance, and the quality of those schemes or methods. | Detailed pilot study protocol informed by formative work, including structured observations and focus groups, approved by Duke Health Institutional Review Board in the United States and University of San Martin De Porres in Peru. | Structured observations, focus groups, pilot study protocol |
| B | Engaging | Attracting and involving appropriate individuals in the implementation and use of the intervention through a combined strategy of social marketing, education, role modeling, training, and other similar activities. | Stakeholders involved in formative work will be given training in use of the mIVAA system during pilot implementation. La Liga leadership has participated in planning conversations with study team as protocol and mIVAA App have been developed.  La Liga social workers coordinate with municipalities to coordinate screening campaigns and also train local women as health promotors to inform women in the communities about screening campaigns. Social workers also utilize social networks (Facebook live e.g.) to inform and engage the local community in screening campaigns. Once women come to the mobile units mIVAA is introduced as part of informed consent. | Focus groups, pilot study protocol, unpublished data form study coordinator |
| 1 | Opinion Leaders | Individuals in an organization who have formal or informal influence on the attitudes and beliefs of their colleagues with respect to implementing the intervention. | For the purpose of this work this role is filled by formally appointed internal leaders including a full-time study coordinator embedded as La Liga staff. | Pilot study protocol |
| 2 | Formally Appointed Internal Implementation Leaders | Individuals from within the organization who have been formally appointed with responsibility for implementing an intervention as coordinator, project manager, team leader, or other similar role. | Full time study coordinator is embedded at La Liga as the internal implementation lead with the planned hiring of additional research assistants to support data collection. Midwife manager and Director of Operations also involved in planning implementation and operations. | Pilot study protocol |
| 3 | Champions | Individuals who dedicate themselves to supporting, marketing, and ‘driving through’ an implementation, overcoming indifference or resistance that the intervention may provoke in an organization. | Same structures in place as formally appointed implementation leaders. | Pilot Study Protocol |
| 4 | External Change Agents | Individuals who are affiliated with an outside entity who formally influence or facilitate intervention decisions in a desirable direction. | Local Principal Investigator at La Liga is a well-respected leader and influencer at La Liga. Co-investigator from Medical Innovation and Technology (MI&T) is closely involved in decision-making on mIVAA software and its design. | Pilot Study Protocol |
| C | Executing | Carrying out or accomplishing the implementation according to plan. | Will be assessed as intervention fidelity and compliance during the pilot implementation | Pilot study data (forthcoming) |
| D | Reflecting & Evaluating | Quantitative and qualitative feedback about the progress and quality of implementation accompanied with regular personal and team debriefing about progress and experience. | Will be assessed through qualitative and quantitative data collection from study participants in the pilot study. | Pilot study data (forthcoming) |

*Indicates constructs underwhich we identified barriers to implementation

Mueller JL, Lam CT, Dahl D, et al. Portable Pocket colposcopy performs comparably to standard-of-care clinical colposcopy using acetic acid and Lugol’s iodine as contrast mediators: an investigational study in Peru. *BJOG An Int J Obstet Gynaecol*. 2018;125(10):1321-1329. doi:10.1111/1471-0528.15326

Vasudevan L, Stinnett S, Mizelle C, et al. Barriers to the uptake of cervical cancer services and attitudes towards adopting new interventions in Peru. *Prev Med Reports*. October 2020:101212. doi:10.1016/j.pmedr.2020.101212

Mezarina LR, Silva-Valencia J, Escobar-Agreda S, et al. Need for the development of a specific regulatory framework for evaluation of mobile health apps in Peru: Systematic search on app stores and content analysis. *JMIR mHealth uHealth*. 2020;8(7):e16753. doi:10.2196/16753

Congress of the Republic of Peru: Law No 30421: National Telemedicine Framework, 2016

**Table S3: Additional Barriers to Implementation**

| **Issue** | **Supporting Quote** | **Reason we did not address** |
| --- | --- | --- |
| Time to sterilize speculums between patients | “The nursing technician commented that before sterilizing the speculums, it was necessary to pre-heat the sterilizer for an hour and then when the speculums are placed there, they stay in for an hour.” | Speculum sterilization is an ongoing issue not specific to or addressed in this study. Issues with time for speculum sterilization will be captured in pilot study data. |
| Cost and distance of follow-up colposcopy | “The colposcopy, because when we give the patient her appointment, she asks how much it costs, how much do I have to pay, if I have to pay or not, because the majority, as we go to zones with low resources, they think is free and when you tell her about costs, often feel discouraged.” | Not addressed in the study. Every woman who is screened in the mobile unit receives a 20% discount for follow up service. Money for transportation is provided based on need. Pilot study will track patient-side barriers to follow-up. |
| Patient phone numbers may be incorrect or out of service | “Patients do not always provide her real information. Women don’t give phone numbers, a reference number, sometimes they don’t know their own phone number, then how we are going to inform them?” | Not addressed in the study. Study will document instances where participants’ phones were not reached. |
|  | “Coordination and management of community education and monitoring of patients through home visits that [did not] answer the follow-up calls of [name] or that the number that the patients have provided is incorrect or is out of service.” | Not addressed in the study. Study will document instances where participants’ phones were not reached. |
| Staff reliability | “Midwife arrived to the mobile unit and had to organize and clean up the mobile unit, since nurse technician arrived 40 minutes late.” | Staff reliability and unplanned absences are universal issues. Issues with staffing will be captured in pilot study data. |
